# Hole-Type Spacers for More Stable Shale Gas-Produced Water Treatment by Forward Osmosis

**DOI:** 10.3390/membranes11010034

**Published:** 2021-01-03

**Authors:** Jawad AlQattan, Youngjin Kim, Sarah Kerdi, Adnan Qamar, Noreddine Ghaffour

**Affiliations:** 1 Water Desalination and Reuse Center (WDRC), King Abdullah University of Science and Technology (KAUST), Thuwal 23955-6900, Saudi Arabia; jawad.alqattan@kaust.edu.sa (J.A.); kyuksh@gmail.com (Y.K.); adnan.qamar@kaust.edu.sa (A.Q.); noreddine.ghaffour@kaust.edu.sa (N.G.); 2 Department of Environmental Engineering, Sejong Campus, Korea University, 2511, Sejong-ro, Jochiwon-eup, Sejong-si 30019, Korea

**Keywords:** forward osmosis, spacer design, shale gas produced water, physical cleaning, fouling and scaling

## Abstract

An appropriate spacer design helps in minimizing membrane fouling which remains the major obstacle in forward osmosis (FO) systems. In the present study, the performance of a hole-type spacer (having holes at the filament intersections) was evaluated in a FO system and compared to a standard spacer design (without holes). The hole-type spacer exhibited slightly higher water flux and reverse solute flux (RSF) when Milli-Q water was used as feed solution and varied sodium chloride concentrations as draw solution. During shale gas produced water treatment, a severe flux decline was observed for both spacer designs due to the formation of barium sulfate scaling. SEM imaging revealed that the high shear force induced by the creation of holes led to the formation of scales on the entire membrane surface, causing a slightly higher flux decline than the standard spacer. Simultaneously, the presence of holes aided to mitigate the accumulation of foulants on spacer surface, resulting in no increase in pressure drop. Furthermore, a full cleaning efficiency was achieved by hole-type spacer attributed to the micro-jets effect induced by the holes, which aided to destroy the foulants and then sweep them away from the membrane surface.

## 1. Introduction

Among osmosis-driven membrane separation processes, forward osmosis (FO) research has seen a significant impulse in the past few years [1,2,3]. FO uses a semi-permeable membrane for separating two solutions with different concentrations [4]. Water molecules migrate from the feed solution (FS) having a low concentration to the draw solution (DS) having a high concentration. The transport of water molecules is purely driven by the osmotic pressure difference which is created across the membrane by the concentration gradient of FS and DS cross-flowing liquids [5]. This aids in eliminating the need for applying hydraulic pressure, resulting in high fouling reversibility on FO membrane and ensuring longer membrane usability [6,7]. Consequently, the use of a good DS solution is highly desirable to achieve low energy requirements and enhanced FO performance [8]. The ideal DS should be easily regenerated at low cost, produce simultaneously high water flux and osmotic pressure, and generate low reverse draw solute flux and concentration polarization.

Produced water contains high total dissolved solids (TDS) levels, which include hydrocarbons, heavy metals, and chemical additives that are harmful to human and marine organisms if they are discharged without any treatment [9,10]. Although produced water is potent in terms of intrinsic/extrinsic inorganic fouling [11], it has a higher salinity than the seawater [12,13], thereby more suitable for FS in FO processes. Hickenbottom et al. [14] have used commercial cellulose triacetate (CTA) membrane to treat produced water by FO using sodium chloride (NaCl) solution (260 g/L) as DS. It was observed that the dilution of DS led to reducing the flux rate over some time. However, it was successfully demonstrated that FO is an effective technique to treat produced water. Hutchings et al. [15] treated produced water to be reused in hydraulic fracturing operations. NaCl concentration of 26% *w*/*w* as DS and produced water with TDS of about 5000 mg/L as FS were utilized in the study. The wastewater of around 70% was successfully reclaimed, which clearly demonstrated that FO has high potential in extracting pure water from the produced water.

Membrane (bio)fouling is inevitable in all membrane processes and it affects the filtration performance in terms of permeate flux production [16,17,18]. Moreover, in pressure-driven membrane processes such as reverse osmosis (RO) and ultrafiltration (UF), (bio)fouling reduces the filtration channel porosity resulting in a significant increase of applied hydraulic pressure, thereby raising the energy requirements of filtration processes [19]. Holloway et al. studied the comparison of membrane fouling between FO and RO processes [20]. They found that the water flux decline was more rapid in RO compared to FO. Moreover, Lee et al. [3] investigated the organic fouling in both FO and RO. They reported that FO could remove organic fouling when the flow velocity was increased, whereas no change in organic fouling amount was monitored in the case of RO process. Furthermore, membrane orientation (i.e., active layer facing FS (AL-FS) or active layer facing DS (AL-DS)) can influence the fouling tendency [21,22]. When AL-DS mode is employed, the fouling in the support layer (facing the FS) could be high and lead to a decrease of the support layer porosity [23]. Promising recent studies focused on the optimization of the membrane materials (e.g., graphene-based membranes) to generate more fouling resistance associated with higher water permeability and selectivity for enhanced desalination systems [24,25,26].

FO has less compact fouling due to the high membrane fouling reversibility which is caused by the lack of hydraulic pressure [27]. Mi et al. [28] studied the fouling reversibility of FO membrane by using sodium alginate as an organic foulant model. They concluded that simple physical cleaning could achieve quasi-full fouling reversibility. Another study realized by Zhan et al. [29] investigated the fouling reversibility in FO by using wastewater as FS. They demonstrated that FO could be used to treat high potential fouling feed. Kim et al. [30] studied the treatment of coal seam gas (CSG) produced water by using fertilizer-dawn FO (FDFO) to produce a nutrient-rich solution for irrigation. They concluded that the RO-FDFO hybrid process was an effective method for treating CSG produced water compared to FDFO and RO. Despite the high fouling reversibility, FO membranes suffered from membrane scaling due to the high content of inorganics. Therefore, an effective method for membrane fouling mitigation is still challenging for the treatment of produced water.

Feed spacers aid not only to support the membrane layers inside the filtration module but also to mitigate the concentration polarization (CP), resulting in lower membrane fouling and enhanced water flux production due to the promotion of unsteadiness/turbulence in the channel [19,31]. Research studies have been done to investigate the effect of spacer design on enhanced filtration performances and membrane fouling mitigation [32,33,34,35,36,37,38,39,40,41,42,43,44,45]. Zhang et al. [35] have investigated the effect of spacer integration in FO system in terms of CP. The authors demonstrated that a spacer can mitigate the internal CP (ICP) when it is inserted in the feed side. On the other hand, they revealed that external CP (ECP) and ICP could be simultaneously decreased when spacers are placed on both feed and draw sides of the membrane. Zou et al. [46] showed the influence of feed spacers in FO by using microalgae as foulant. They concluded that spacers could help to improve the initial water flux and reduce the membrane fouling. Furthermore, research studies demonstrated that the geometry of the spacers could play an essential role in enhanced membrane filtration performances. However, the incomprehensive optimization of various spacer geometric parameters (such as spacer design, filament shape, filament thickness, and internal strand angle) is so far a key challenge in water treatment processes [47]. Haaksman et al. [48] proved that a lower CP could be obtained by alternating spacer filament thicknesses due to the generation of high shear stress on membrane surface. Moreover, the spacer orientation and filament shape were found to have a significant influence on the fouling and hydrodynamic conditions [32,49]. Neal et al. [50] studied the effect of spacer orientation (0°, 45°, and 90° to the flow direction) in the filtration channel on the critical flux by investigating the particle deposition on the spacer. They concluded that 0° provided the most significant improvement in critical flux whereas 90° gave the least. Although the spacer can help to improve the membrane performance, particles might be deposited in the dead zones (where little/no fluid is flowing) behind the spacer filament intersections and lead consequently to diminish the overall performance of the filtration system. Kerdi et al. [33] examined the performance of perforated feed spacers in a cross-flow UF system by varying the inlet feed velocity magnitude. The authors exhibited that a great improvement of the filtration performance was particularly achieved when the holes are created at the spacer filament intersections (hole-type spacer). In the present work, the same hole-type spacer design already reported by Kerdi et al. [33] for ultrafiltration was evaluated in FO process by varying the feed and draw solutions. This spacer design with holes at filament intersections shows its ability to locally provide unsteadiness inside the filtration channel that can effectively clean the membrane surface. Moreover, this type of spacer has the potential to enhance the permeate water flux and reduce the reverse salt flux (RSF) and fouling deposition on spacer filaments and FO membrane surface. Therefore, the overall FO performance could be improved without the need for frequent cleaning. Besides, as FO is known to treat high salinity produced water [14,15,51], the treatment of shale gas produced water (SGPW) is further tested in this study to evaluate the promising performance of hole-type spacer in FO process. The main objective of this study is to evaluate the performance of hole-type spacer design in SGPW treatment [51,52,53]. In the present study, FO process was firstly examined in presence of hole-type spacer in terms of water flux, reverse solute flux (RSF), and reverse solute flux selectivity (RSFS) by varying DS and FS concentrations. Secondly, its filtration performance was evaluated and compared to standard spacer (without holes in spacer filaments) and no spacer cases. Finally, by using SGPW as FS, the flux decline, accumulation of fouling on membrane surface, pressure drop increase, and physical cleaning efficiency were examined in the absence and presence of spacers (hole-type and standard spacers).

## 2. Materials and Methods

### 2.1. FO Membrane and Draw Solution

Commercially available cellulose triacetate (CTA) FO membrane embedded in a woven polyester mesh was used in all experiments. This membrane was provided by Hydration Technology Innovations (HTI, Albany, OR, USA). Water and solute permeability coefficients and structural parameters of the membrane are presented in Table S1 in the Supplementary Materials. Detailed characteristics are reported elsewhere [54,55]. The membrane coupons were immersed in Milli-Q water and stored at 4 °C. Sodium chloride (NaCl) provided from Fisher Scientific (Pittsburgh, PA, USA) was dissolved in Milli-Q water at different concentrations (0.6 M, 1.2 M, 1.8 M, and 5 M) to be utilized as DS in the present study.

### 2.2. Synthetic Shale Gas Produced Water (SGPW)

Synthetic SGPW was employed as FS to evaluate the hole-type spacer performance with a feed containing high TDS. The composition of SGPW was adopted according to the typical constituents used in Marcellus shale gas (USA) [56]. This solution was prepared by dissolving all chemical products summarized in Table 1 in Milli-Q water. All used chemicals had powder form and provided by Sigma Aldrich (St. Louis, MO, USA).

### 2.3. Feed and Draw Spacers

**Figure 1:**
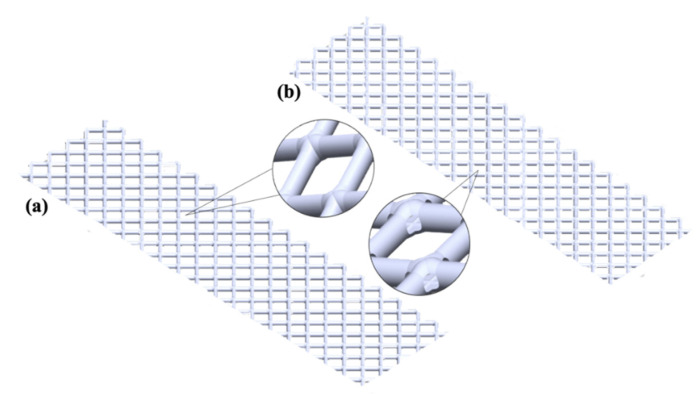
CAD models for (**a**) standard spacer and (**b**) hole-type spacer.

Standard and hole-type spacers used in this study were designed by computer-aided design (CAD) in SolidWorks software (Dassault System Solid-Works Cooperation, Waltham, MA, USA) and were then manufactured by utilizing 3D-printing technology (MiiCraft 125, Version 3.4.5, MiiCraft Inc., New York, NY, USA). This 3D-printer allows printing spacers through UV polymerization of liquid resin (BV-007acrylate monomer, MiiCraft Inc., New York, NY, USA) with a high resolution (25 µm printing layer). Figure 1 shows CAD models of the standard and hole-type spacers where the same geometric parameters were kept for both spacers except the creation of holes (diameter of 0.5 mm) at the filament intersections of hole-type spacer design. The thickness of both spacers was 1.8 mm. More detailed information concerning the geometric parameters of the spacer design was described elsewhere [33].

### 2.4. Lab-Scale FO Experiments

Experiments were carried out to evaluate the hole-type spacer performance in FO process in terms of water flux, RSF, and RSFS by using a customized lab-scale FO unit. Two variable speed pumps (Cole-Parmer, Vernon Hills, IL, USA) were used to circulate FS and DS solutions in a closed-loop, which led to batch operation. To measure the weight change over time, the DS tank was placed on a digital balance having high sensitivity (MS204, Mettler Toledo, Columbus, OH, USA). All balance readings were continuously recorded through a data acquisition system (LabView, version 14, National Instruments, Austin, TX, USA) at a time interval of 1 min to determine the evolution of water fluxes over time. CTA membrane was installed in a custom-made cross-flow cell having symmetric flow channels at both sides of the membrane. The measured membrane active area was 60 mm × 15 mm. A conductivity meter (Hach, Düsseldorf, Germany) was connected to the FS tank to continuously measure the TDS of FS at a time interval of 15 min to calculate the RSF.

FO experiments were carried out under AL-FS mode where the active layer side was facing the FS. For each experiment, a new membrane coupon was placed in the flow cell and spacers were installed on both feed and draw sides of the membrane. The flow rates in FS and DS were set at 300 mL/min for all experiments, and the volume of both solutions was set to be 1 L with a temperature of 25 °C throughout the FO tests. In the case of synthetic SGPW treatment, the experiment was performed until collecting 180 mL of permeation through FO membrane. It is relevant to emphasize here that each experiment was duplicated and the averages of resulted water fluxes were plotted in the study.

### 2.5. Theoretical Analysis

As shown in Equation (1), the water flux was calculated from the volume change of the DS divided by the membrane area and time [57]:

(1) $$J_{w}=\frac{\left(V_{Draw_{f}\;}-\;V_{Draw_{i}\;}\right)\;}{\Delta t*A_{m}}$$

where $J_{w}$ is the water flux (LMH, L/m^2^/h), $V_{Draw_{f}\;}\mathrm{and}\;V_{Draw_{i}}$ are the final and initial volumes of DS (L), respectively, $A_{m}$ is the active membrane area (m^2^), and Δ*t* is the time needed to produce $J_{w}$ (h). The RSF was resulted from the increase of TDS in FS and was calculated following Equation (2) [8]:

(2) $$J_{s}=\frac{(C_{t}V_{t})-\left(C_{0}V_{0}\right)}{\Delta t*A_{m}}$$

where $J_{s}$ is the reverse solute flux (gMH, g/m^2^/h), $C_{t}$ and $V_{t}$ are, respectively, the salt concentration (g/L) and feed volume (L) over a predetermined time *t* (h), whereas $C_{0}$ and $V_{0}$ are the initial concentration (g/L) and feed volume (L), respectively.

To evaluate the performance of the hole-type spacer, the solute resistivity (K), which is the measurement of the solute diffusion into the membrane support layer, was further determined by using the water flux equation developed by McCutcheon and Elimelech [58] (Equation (3)). The structural parameter was calculated by using solute resistivity and diffusivity, as shown in Equation (4) [54,59]:

(3) $$J_{w}=A\left[\pi_{D\;}\exp\left(-J_{w}K\right)-\pi_{F\;}\exp\left(\frac{J_{w}}{k}\right)\right]$$

(4) $$K=-\frac{\ln\left[\frac{J_{w}}{A\;\pi_{D}}\right]}{J_{w}}=\frac{S}{D}$$

where A is the water permeability coefficient of the membrane (LMH/bar), K is the solute resistivity (s/m), $\pi_{D\;}$ and $\pi_{F\;}$ are the osmotic pressures of the bulk DS and FS (bar), respectively,  k is the mass transfer coefficient (m/s), and D is the diffusivity (m^2^/s). It is relevant to highlight that in the case of Milli-Q water used as FS, the osmotic pressure of FS was almost equal to zero, which was neglected and helped to simplify Equation (3) into Equation (4). k values associated with calculated structural parameters were further served to calculate the theoretical water fluxes as shown in Equation (3). The theoretical *k* values were determined by calculating the channel porosity (*ε*) and the average effective velocity (*U_ave_*) (Equations (5)–(8)) were followed to calculate Reynold (*Re*) and Schmidt (*Sc*) numbers, respectively [60,61]. Moreover, the hydraulic diameter (*d_h_*) and Sherwood number (*Sh*) were calculated by using the equations listed in Table 2. Sherwood number, hydraulic diameter, and diffusivity (*D*) were then employed to determine *k*, as shown in Equation (9) [58]:

(5) $$\varepsilon =1-\frac{V_{sp}}{V_{total}}$$

(6) $$U_{ave}=\frac{Q}{w\;h\;\varepsilon }$$

(7) $$Re=\;\frac{U_{ave}d_{h}}{v}$$

(8) $$Sc=\frac{v}{D}$$

(9) $$k=\frac{Sh\;D}{d_{h}}$$

where *ε* is the porosity, $V_{sp}$ and $V_{total}$ are the spacer volume and the total volume of the channel (m^3^), respectively, $U_{ave}$ is the average effective velocity (m/s), Q is the volumetric feed flow rate (mL/min), w is the width of the flow channel (m), h is the height of the flow channel (m), Re is Reynold number, $d_{h}$ is the hydraulic diameter (m), v is the kinematic viscosity (m^2^/s), Sc is Schmidt number, $S_{SP}$ is the surface of spacer (m^2^), and l is the length of the flow channel (m).

RSFS is defined as the ratio of the water flux to the RSF as presented in Equation (10) [63]:

(10) $$RSFS=\;\frac{J_{w}}{J_{s}}=\left(\frac{A}{B}\right)nR_{g}T$$

where B is the solute permeability coefficient of the membrane (LMH), n is the number of dissolved species formed in the draw solute (e.g., 2 for NaCl), $R_{g}$ is the ideal gas constant (L∙bar/K∙mol), and *T* is the absolute temperature (K).

### 2.6. Membrane Surface Characterization

The morphology of the fouling layer developed on the membrane surface was characterized by using scanning electron microscopy (SEM, Zeiss supra, Carl Zeiss AG, Oberkochen, Germany). The sample was prepared for SEM analysis by soaking the fouled membrane in Milli-Q water for a couple of seconds in order to remove residues of FS and DS solutions, and then it was kept for one day to be dried. Energy-dispersive X-ray spectroscopy (EDX) analysis was carried out to identify the various compositions/dispersion of the foulant accumulated on membrane surface. SEM imaging and EDX spectroscopy were conducted with an accelerating voltage of 5 kV and 20 kV, respectively.

### 2.7. Pressure Drop Measurement and Physical Cleaning

The pressure drop for the feed flow channel was measured before and after SGPW treatment by using a differential pressure transmitter (model PX5200-400WUDI, Omega, Norwalk, CT, USA). After SGPW treatment, simple hydraulic flushing for physical cleaning was carried out by increasing the flow rate up to three times (900 mL/min) for 15 min. The cleaning efficiency was then assessed by calculating the flux recovery which represents the ratio of water fluxes produced before and after physical cleaning according to Equation (11) [64]:

(11) $$Flux\;recovery=\;\frac{J_{wc}}{J_{wi}}*100\%$$

where $J_{wc}$ and $J_{wi}$ are the water fluxes produced after and before physical cleaning, respectively.

## 3. Results and Discussion

### 3.1. Evaluation of Hole-Type Spacer Performance in FO Process

**Figure 2:**
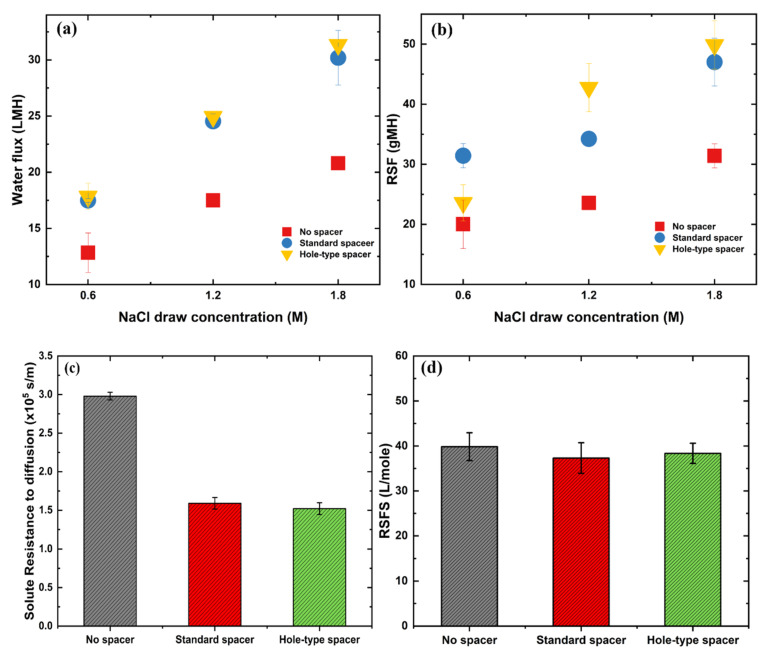
Experimental FO results in absence/presence of spacers (hole-type and standard spacers): (**a**) water flux, (**b**) reverse solute flux (RSF), carried out by using FS as Milli-Q water and various NaCl DS concentrations and calculated based on Equation (1) and Equation (2), respectively, (**c**) solute resistance, and (**d**) reverse solute flux selectivity (RSFS) of the membrane calculated based on Equation (4) and Equation (10), respectively.

To evaluate the performance of the hole-type spacer design in FO system, various experiments were carried out by using Milli-Q as FS and NaCl having different concentrations (0.6 M, 1.2 M, and 1.8 M) as DS. The resulted filtration performance was assessed relative to the performances obtained by using a standard spacer design and without any spacer integration in the filtration channel. As shown in Figure 2a, regardless of NaCl concentration in DS, both spacers improved the water flux production compared to that produced in absence of spacer (*J_w_* = 13 LMH). In case of low NaCl concentration (0.6 M), the hole-type spacer produced slightly higher water flux (*J_w_* = 18 LMH) than standard spacer (*J_w_* = 17 LMH). When the concentration of DS was increased to 1.2 M of NaCl, 43% and 40% of permeate flux enhancements were respectively acquired for hole-type (*J_w_* = 25 LMH) and standard (*J_w_* = 24 LMH) spacers relative to no spacer experiment (*J_w_* = 17 LMH). By using a DS with the highest NaCl concentration (1.8 M), produced water fluxes were found greater to be 21 LMH, 30 LMH, and 31 LMH in case of no spacer, standard and hole-type spacers, respectively. Hence, at this DS concentration range, a relatively highest flux enhancement (51%) was realized in presence of hole-type spacer relative to the absence of spacer in the channel.

Similar to water flux, the presence of spacers led to an increase of RSF values for all tested DS concentrations (Figure 2b). At low DS concentration (0.6 M), hole-type spacer revealed 25% less RFS value (*J_s_* = 24 gMH) compared to standard spacer (*J_s_* = 31 gMH). However, this behavior was reversed at high DS concentrations (1.2 M and 1.8 M) and the RFS improvement was observed more pronounced at DS concentration of 1.2 M of NaCl. At this DS concentration, RFS value was found to be 25% greater for hole-type spacer compared to standard spacer (*J_s_* = 34 gMH and 42 gMH for standard and hole-type spacers, respectively).

The aforementioned results confirmed that the insertion of spacers can improve the filtration performance in FO systems due to the turbulence/unsteadiness induced in the channel. Moreover, it has been demonstrated that the presence of spacer in the feed side helped to reduce ECP on membrane surface [34], thereby enhancing the water flux production. Our results revealed that the hole-type spacer aided to slightly increase the water flux production compared to the standard spacer though that higher flux improvement was expected. It has been predicted that the creation of holes at the spacer filament intersections might contribute to establishing additional fluid flow ways inside the channel, resulting in a greater improvement in the flux production.

Theoretically and according to Equation (3), spacers cannot improve FO performance in the case of Milli-Q water used as FS. Consequently, it can be assumed that spacers help to decrease solute resistance (*K*) in the support layer and mitigate ICP [65,66]. *K* parameters were then calculated based on Equation (4), and the resulted values were presented in Figure 2c. The results show that the highest *K* value was obtained in absence of spacer (*K* = 3 × 10^5^ s/m), followed by standard spacer (*K* = 1.60 × 10^5^ s/m) and then hole-type spacer (*K* = 1.50 × 10^5^ s/m). In presence of spacers, a reduction in *K* parameters was detected to reduce ICP within the support layer which, in its turn, became less severe than in the absence of spacer. Therefore, an enhancement in the effective concentration gradient was achieved resulting in an improvement of FO performance. It is relevant to highlight here that hole-type spacer showed a lower *K* value than standard spacer despite the lower cross-flow velocity (i.e., 0.38 m/s and 0.54 m/s, respectively) (Figure 2c). It can be concluded that the creation of holes (micro-jets) in the spacer design allowed the flowing of even within the filament intersections, which led to promote stronger turbulence and effectively mitigate ICP formation.

The draw solute in FO can be lost through RSF which is defined as the permeation of the draw solutes through the membrane from DS solution into FS solution. Thus, the membrane ability to minimize the draw solute loss is referred to RSFS [67]. RSFS (volume of the produced water per the lost mass of draw solute) was then calculated following to Equation (10) to further investigate the impact of spacer on FO performances. As shown in Figure 2d, no significant change in RSFS values was observed for the three tested cases. It could be inferred that hole-type spacer was not able to control the loss of draw solutes through the membrane. Nonetheless, these obtained results proved that no damage to the membrane surface was occurred due to the presence of the spacer.

**Figure 3:**
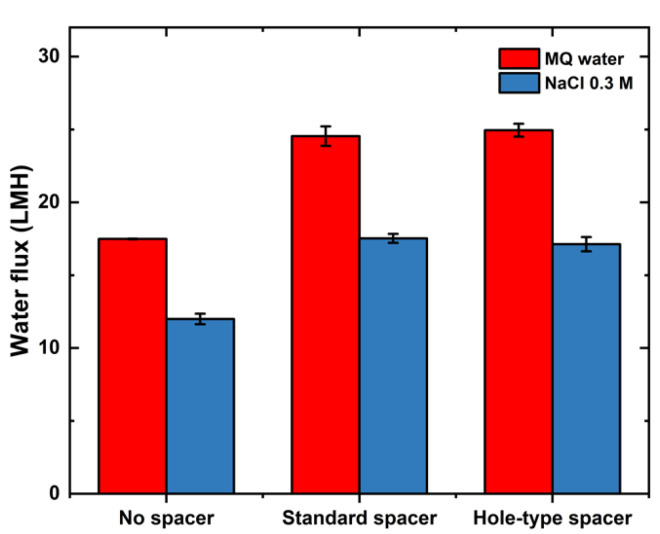
Water fluxes produced by using NaCl (1.2 M) as DS and different FS concentrations (Milli-Q water and NaCl (0.3 M)) in FO system.

In an attempt to assess the impact of spacers on ECP, a set of experiments was carried out by using sodium chloride solution (0.3 M) as FS instead of Milli-Q water. In these experiments, NaCl solution having a concentration of 1.2 M was used as DS. The obtained results are summarized in Figure 3. 

A decrease in water flux production was observed, which was attributed to the effective concentration gradient reduced by increased FS concentration. To confirm the effect of spacers on ICP, water flux was simulated based on Equation (3) and the values were presented in Figure S1 (presented in the Supplementary Materials). The Reynolds numbers were calculated for all experiments and reported to be 649, 705, and 711 for no spacer, standard spacer and hole-type spacer, respectively. No significant difference in experimental and numerical water flux values (−11.4%, −3.4%, and 1% for no spacer, standard spacer, and hole-type spacer, respectively) was detected. This finding confirms that spacers aid to mitigate ICP formation in the support layer by promoting more turbulence in the filtration channel.

### 3.2. Evaluation of Hole-Type Spacer Performance for Synthetic SGPW Treatment in FO

#### 3.2.1. Water Flux Decline

**Figure 4:**
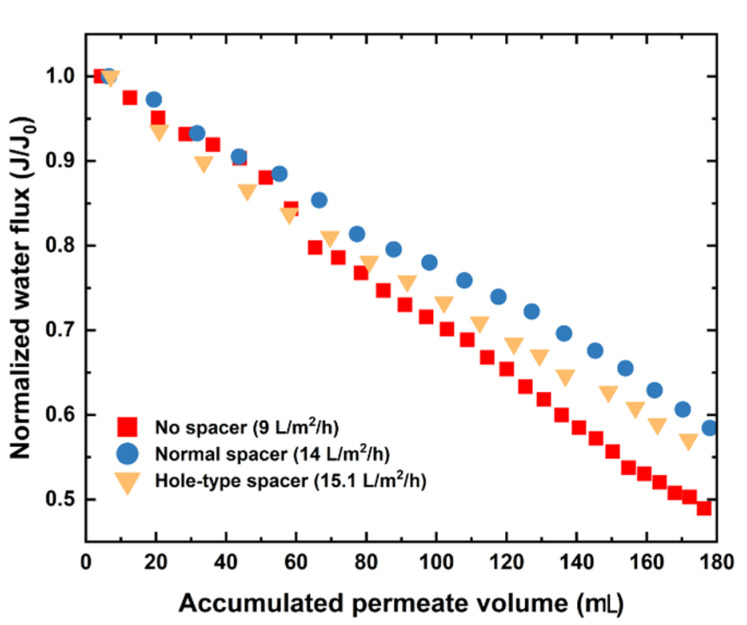
Normalized water flux as a function of accumulated permeate volume, NaCl (5 M) is used as DS for all experiments.

To realize SGPW treatment, the DS concentration used in FO experiments was selected to be 5 M NaCl in order to produce enough water flux. Figure 4 depicts the normalized water flux (ratio of water flux produced at time t (*J_w_*) to its corresponding initial value (*J_w0_*)) versus accumulated permeate volume for various experimental cases. Lower initial water flux was produced (*J_w0_* = 9 LMH) in absence of spacer compared to those produced in presence of hole (*J_w0_* = 15 LMH) and standard (*J_w0_* = 14 LMH) spacers. In these operating parameters, the highest water flux was further obtained with hole-type spacer, which was explained by the presence of micro-jets in the design helping to generate more turbulent eddies (more breakdown vortices) at the downstream of spacer filaments. As shown in Figure 4, a decline in water flux was observed during synthetic SGPW treatment for all cases. This decline might be attributed to the membrane fouling layer formed on the membrane surface. Unlike our prediction, hole-type spacer had a higher flux decline than standard spacer. It is recognized that a higher initial water flux (higher permeation drag force) might aggravate the fouling development on membrane surface [22], which results in a higher flux decline explaining the severe flux decline for hole-type spacer case. It is useful to emphasize here that the highest flux decline was observed in absence of spacer in the filtration channel, which confirmed that the presence of spacer helped to recover the flux production.

Furthermore, the Reynolds numbers were calculated and reported to be 547, 590, and 600 for no spacer, standard spacer, and hole-type spacer, respectively. The experimental water flux values were found considerably consistent with the numerical values as shown in Figure S2 presented in the Supplementary Materials (−6.2%, 17.3%, and 10.9% for no spacer, standard and hole-type spacers, respectively). Spacers have then the potential to produce higher water fluxes, which could explain the formation of lower ICP in the membrane support layer.

#### 3.2.2. Fouling Characterization on Membrane Surface

**Figure 5:**
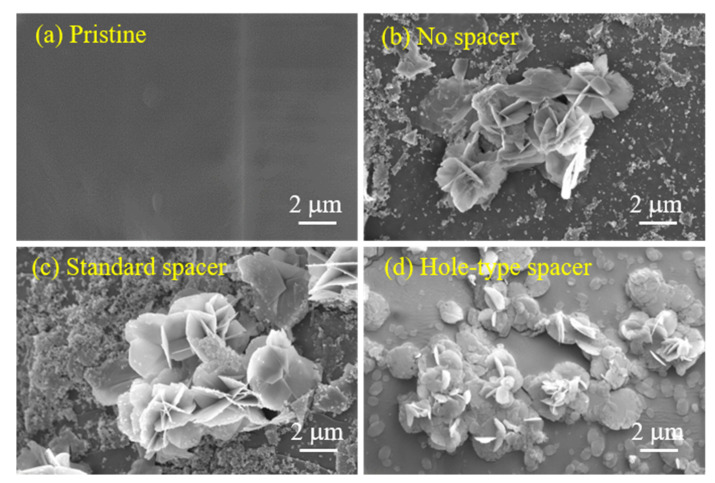
SEM images of the active layer surfaces of (**a**) pristine membrane, (**b**) membrane with no spacer, and membranes equipped with (**c**) standard and (**d**) hole-type spacers.

Scale formation is a complex phenomenon that could affect the performance of membrane technologies [68]. Surface and bulk crystallization are the major factors inducing and scaling fouling in membrane systems. The surface crystallization occurred due to the significant growth of scales on membrane surface causing its blockage leading to a flux decline. However, when the crystallization of particles takes place in the bulk phase followed by their deposition on membrane surface, the bulk crystallization occurs resulting in a further decline in water flux [69]. To characterize the scale formed on the surfaces of FO membrane used for SGPW treatment, SEM images were performed on their active layer surfaces and the scanned images are shown in Figure 5.

SEM images demonstrated that scale formation occurred on membrane surface for all cases (Figure 5a–d). In agreement with other studies [69,70], the scaling observed on the membrane surface of no spacer (Figure 5b) and standard spacer (Figure 5c) cases were attributed to the barite crystals deposition, which is subsequently confirmed by EDX analysis (Figure 5). The composition of SGPW led to the formation of barium sulfate (BaSO_4_), which has low solubility in water resulting in its scaling on membrane surface [71]. In the case of hole-type spacer, SEM image (Figure 5d) revealed smaller particles spreading over the membrane area. These particles were broken down due to the high shear force induced by the micro-jets created by the presence of holes in the spacer design. As a result, scaling was found covering a wide area of membrane surface (hole-type spacer). This insight could explain the higher flux decline obtained with this spacer when compared to the standard spacer (Figure 4). This phenomenon was not observed in earlier study where different types of membrane fouling (i.e., organic and colloidal fouling) were formed on the membrane surface, where hole-type spacer showed less flux decline due to the scouring effect of micro-jets [30].

**Figure 6:**
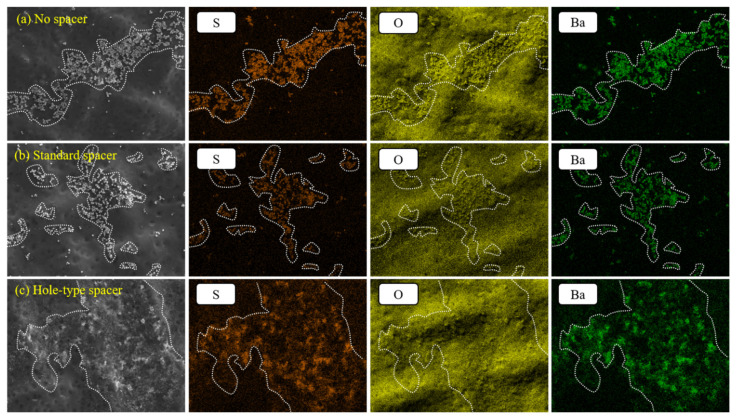
EDX mapping of the active layer surfaces of membranes: (**a**) with no spacer, (**b**) equipped with standard, and (**c**) hole-type spacers.

EDX analysis was further performed to identify the scaling type on various membrane surfaces. For all experimental cases, EDX results exhibited peaks of Ba, S, and O elements demonstrating the presence of BaSO_4_ on the membrane surface. Moreover, sodium chloride was detected as well, which indicated that a reverse diffusion of solutes (NaCl) occurred from the DS solution. To thoroughly identify the scaling on membrane surface, EDX mapping was also carried out and presented in Figure 6. The obtained results proved that the inorganic scaling (O, S, and B elements) was more dispersed and covered a higher area of membrane surface in the case of hole-type spacer confirming again the breakdown of particles due to the higher shear forces and turbulence induced by the spacer holes.

#### 3.2.3. Pressure Drop and Physical Cleaning Efficiency

**Figure 7:**
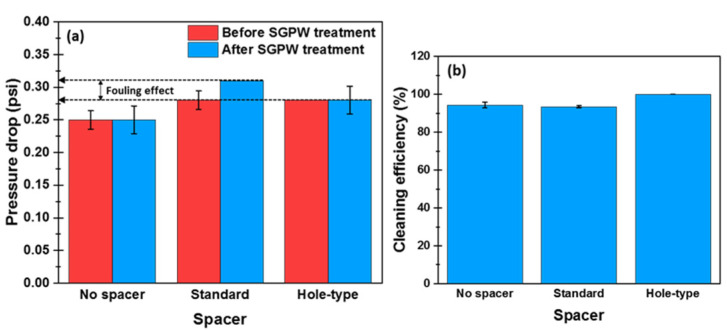
(**a**) Pressure drop measured along the feed filtration channel before and after SGPW treatment, and (**b**) physical cleaning efficiency applied by increasing the inlet cross-flow velocity three times higher than the velocity used throughout the experiments. The cleaning efficiency was calculated based on Equation (11).

To investigate the effect of hole-type spacer on the hydraulic resistance, pressure drop variations of the feed flow channel before and after SGPW treatment were measured for all experimental tests and presented in Figure 7a. In absence of spacer, no change in pressure drop was detected after SGPW treatment and its value was estimated to be 0.25 psi. However, when a spacer was inserted in the filtration channel, the magnitude of pressure drop increased to reach 0.28 psi before SGPW treatment regardless of the type of the inserted spacer. Hence, the insertion of spacers in the feed channel causes obviously this rise in pressure drop along the flow channel [72]. After SGPW treatment, the pressure drop value of standard spacer increased from 0.28 psi to attain 0.31 psi, whereas no pressure drop increase was observed in the case of hole-type spacer. This increase in pressure drop in presence of standard spacer was attributed to the accumulation of fouling material on membrane surface. Contrary, the presence of micro-jets aided to mitigate the foulant accumulation after SGPW resulting in a pressure drop stability in case of hole-type spacer. In conclusion, less pressure drop (thereby, less energy consumption) could be accomplished by using the hole-type spacer.

As FO process utilizes the osmotic pressure gradient as a driving force, it was expected that the membrane fouling layer could be readily removed by a simple physical cleaning such as an application of a strong fluid shear force (an increase of inlet velocity) [7]. Physical cleaning on membrane surface was further performed after SGPW treatment by using Milli-Q water as FS in order to assess the water flux recovery. The physical cleaning efficiency for all experimental cases is presented in Figure 7b. A full flux recovery was achieved in the case of hole-type spacer, whereas 93% and 95% of flux recovery were obtained in the case of standard spacer and the absence of spacer, respectively. Consequently, the scaling on the active layer could be completely removed in the case of hole-type spacer due to its ability to fully improve the physical cleaning efficiency. Therefore, the presence of micro-holes in the spacer design at the filament intersections along with physical cleaning aids to entirely sweep away the fouling layer formed on the membrane surface.

## 4. Conclusions

The performance of hole-type spacer in FO process without and with SGPW treatment was evaluated and compared to the standard spacer and no spacer cases. The results reported in the present study can be summarized as follows:

- Spacers have the ability to enhance the water flux in FO process via the mitigation of ICP. In particular, hole-type spacer at the onset of the experiment aided to increase the water flux slightly more than standard spacer.
- During SGPW treatment, hole-type spacer exhibited higher water flux but severer flux decline. This finding was referred to the presence of holes which aided to break down the scaling to cover a significant area of membrane surface. This was not observed for organic fouling studies reported elsewhere.
- After SGPW treatment, standard spacer exhibited an increase in pressure drop, whereas hole-type spacer had no change in pressure drop. This could effectively minimize the energy consumption in FO modules.
- Hole-type spacer showed higher physical cleaning efficiency (100% of flux recovery) than standard spacer (95% of flux recovery). This finding was attributed to the presence of holes in the spacer filament intersections which helped in readily destroying and removing the scaling layer on membrane surfaces resulting in a more stable FO operation during SGPW treatment.

## Figures and Tables

**Table 1:** Composition of synthetic SGPW.

Compounds	Concentration (g/L)
Sodium bromide (NaBr)	1.54
Barium chloride (BaCl_2_)	5
Sodium sulfate (Na_2_SO_4_)	0.0103
Calcium chloride (CaCl_2_)	27.2
Sodium chloride (NaCl)	110.9

**Table 2:** Equations used to calculate the hydraulic diameter (*d_h_*) and Sherwood number (*Sh*).

	Without Spacer	With Spacer	Reference
Hydraulic diameter	$d_{h}=\frac{2wh}{w+h}$	$d_{h}=\frac{4\;}{\frac{2}{h}+\left(1-\varepsilon \right)\frac{S_{SP}}{V_{SP}}}$	[60,61]
Sherwood number	$Sh=1.85{\left(ReSc\frac{d_{h}}{l}\right)}^{0.33}$	$Sh=0.065Re^{0.875}Sc^{0.25}$	[22,61,62]

## Data Availability

Not applicable.

## Supplementary Materials

The following are available online at https://www.mdpi.com/2077-0375/11/1/34/s1, Table S1. FO membrane characteristics; Figure S1. Experimental and simulated water fluxes produced by using 0.3 M NaCl as FS and 1.2 M NaCl as DS; Figure S2. Experimental and simulated water flux produced by using synthetic produced water as FS and 5 M NaCl as DS.
