# Supplementary material for: Hole-Type Spacers for More Stable Shale Gas-Produced Water Treatment by Forward Osmosis
Source: Membranes (Basel). 2021 Jan 3;11(1):34. doi: 10.3390/membranes11010034 (PMC7824311; doi:10.3390/membranes11010034)
Supplement: Supplementary file 1 [file membranes-11-00034-s001.pdf]

Supplementary Materials

Hole-type spacer for more stable shale gas produced water treatment by forward osmosis

Jawad AlQattan [1], Youngjin Kim [1,2], Sarah Kerdi [1,*], Adnan Qamar [1], Noreddine Ghaffour [1]

[1] King Abdullah University of Science and Technology (KAUST), Water Desalination and Reuse Center (WDRC), Thuwal 23955-6900, Saudi Arabia
[2] Department of Environmental Engineering, Sejong Campus, Korea University, 2511, Sejong-ro, Jochiwon-eup, Sejong-si, Republic of Korea
* Correspondence: sarah.kerdi@kaust.edu.sa

Table S1. FO membrane characteristics.

Parameter	Values
A (LMH/bar)	1.31
B (LMH)	1.80
S (m)	0.000463

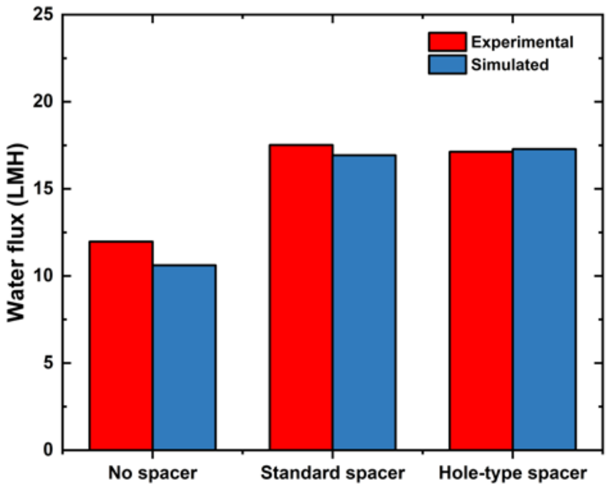

Figure S1. Experimental and simulated water fluxes produced by using 0.3 M NaCl as FS and 1.2 M NaCl as DS.

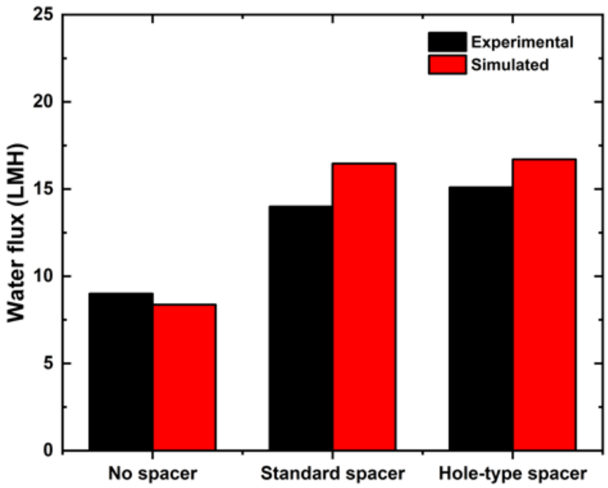

Figure S2. Experimental and simulated water flux produced by using synthetic shale gas produced water as FS and 5 M NaCl as DS.
